# A distribution and taxonomic reference dataset of Geranium in the New World

**DOI:** 10.1038/sdata.2017.49

**Published:** 2017-04-11

**Authors:** Carlos Aedo, Francisco Pando

**Affiliations:** 1 Real Jardín Botánico-CSIC, 28014 Madrid, Spain

**Keywords:** Natural variation in plants, Classification and taxonomy

## Abstract

Geranium L. is a genus of over 350 species distributed throughout most of the world, except in lowland tropical areas. It is the largest genus of the Geraniaceae and is represented in the New World by 137 species. This dataset includes 8,937 records that covers the genus *Geranium* the New World, providing an updated, taxonomically consistent and a sound geographical distribution of the 137 species of Geranium in America. Specimens from 128 herbaria were reviewed. These were supplemented by others collected during nine field trips, which allowed better knowledge of the variability of characters within populations, and refining species distribution ranges. Each record represents a specimen that has been reviewed and in some cases collected by C. Aedo. Accepted scientific name, locality details, distribution status (introduced, native, naturalized, uncertain), geographic coordinates are given for 8,538 (95%) records, and habitat information for 3,952 (44%). All data have been released under a CC-BY license in a standardized format, which enables easy integration with other data, for example through GBIF.org.

## Background & Summary

In pursuit of preparing a comprehensive world monograph of the genus *Geranium*, studies of some groups of *Geranium* from America were carried out as follows:

Species from Canada, Greenland and USA^1,2^.

Sect. *Brasiliensia* R. Knuth from South America^3^.

Sections *Azorelloida* Aedo, Muñoz Garm. & Pando, *Neoandina* Aedo and *Paramensia* R. Knuth^4^.

Sect. *Gracilia* R. Knuth^5,6^.

Sections *Andina* R. Knuth and Chilensia R. Knuth^7^.

Sections *Batrachioidea* W. D. J. Koch and Divaricata Rouy^8^.

Sect. *Trygonium* Dumort^9^.

Sect. *Dissecta* Yeo^10^,

Additionally, several new American species have been published in separate papers^11–18^.

These studies were culminated and completed in a single work^19^, for which additional specimens and natural populations were studied and descriptions, keys and distributions improved. Furthermore, the status and circumscription of these species were re-evaluated and compared with those not previously studied and in many cases emended. The dataset described here brings together the primary data on which these studies were based, and reflects the work done upon them, providing a unified and updated view of the Genus in America available to be used as a base line for further studies. The level of scrutiny carried out on the data makes the dataset readily available for multiple analyses and to be integrated with other data sources. The dataset also contains information reflected in later shorter articles, such as Aedo^20^.

In total, the dataset includes 8,932 records that cover genus *Geranium* the New World, providing an updated, taxonomically consistent and a sound geographical distribution of the 137 species of *Geranium* in America.

**Figure 1:**
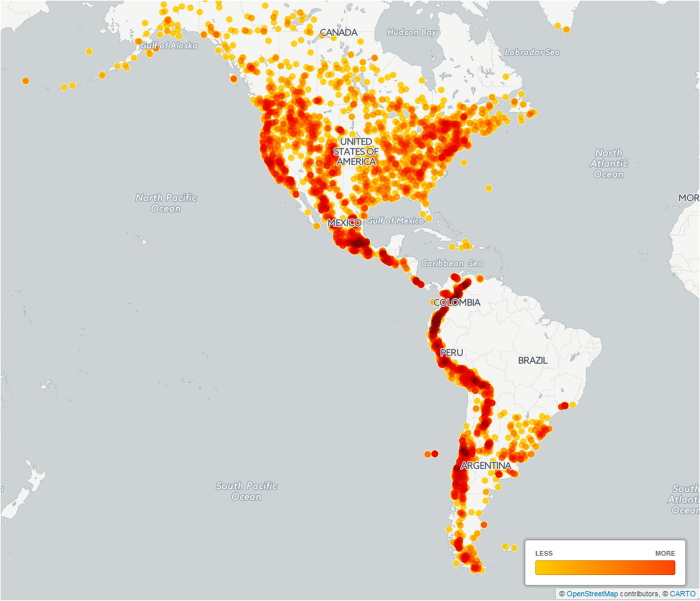
Map of georeferenced specimens. The darker the color, the higher is the record density.

**Figure 2:**
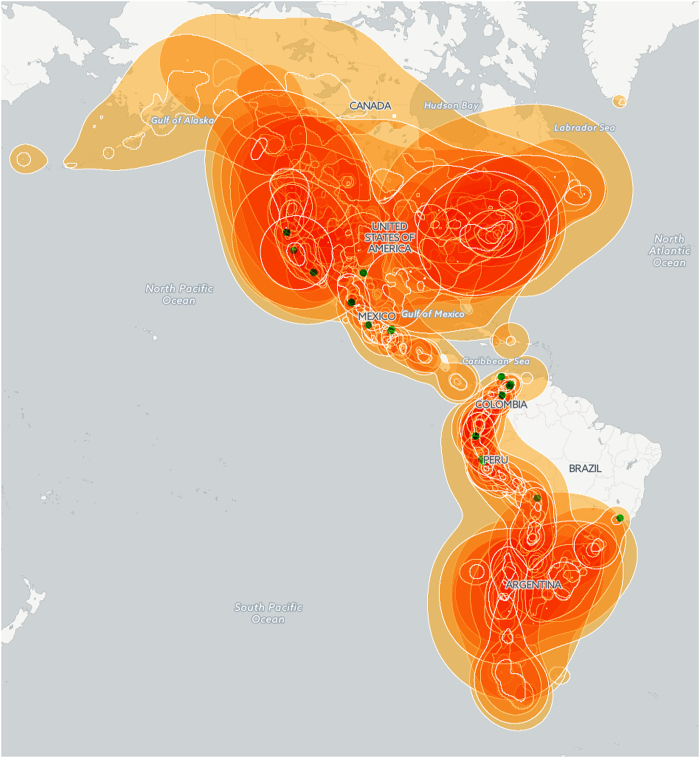
Species richness overview. Map of species range areas generated with ModestR^42^ using a kernel density estimation (cell size=10 minutes, bandwidth=1), not clipped by coastlines. Location of species with too few records to generate areas are visualized as green dots.

Besides the exhaustive taxonomic work done for the monograph, and reflected in the dataset, a detailed revision has been performed of the specimens’ localities and geographic coordinates assigned with a 1-minute accuracy, taking into account knowledge such as species habitat and information provided by the collectors and botanists. Less than two thousand specimens had original coordinates recorded on the labels. In a few cases (396), it has not been possible to georeference the specimens with reasonable certainty, and as a result only 8,487 (95%) of the records have coordinates (see Figs 1 and 2). Herbarium work covered specimens held in 128 collections. Herbaria and number of studied specimens containing the greater number of relevant specimens are listed in Table 1.

Herbarium material was supplemented by specimens collected during nine field trips, which allowed a better knowledge of the variability of characters within populations, and the refining of species distribution ranges. These expeditions took place in Argentina, Bolivia, Chile, Costa Rica, Ecuador, Peru, United States and Venezuela, and yielded 235 herbarium specimens of *Geranium* belonging to 46 species and assessments of natural populations.

Over 5,000 additional herbarium specimens from Africa, Asia, Australia and Europe were examined to determine the variability of non-native species thorough their entire range.

Duplicate specimens kept in different collections have been aggregated into single records.

The potential for this dataset to be widely reused is very high, given the level of taxonomic and geographic scrutiny this dataset encompasses, its completeness, and the fact that *Geranium* is a well-known genus, widely used in horticulture and pharmacy. To facilitate its wider scientific use, the dataset was registered with GBIF.org in order to enable its easy integration with thousands of other biodiversity datasets. GBIF reported that all or part of the dataset contributed to more than 650 data downloads done in the period 14-July and 3-November, 2016, via GBIF.org: http://www.gbif.org/dataset/26d72d3b-4544-4645-aa56-27aa8a669c6f/activity

## Methods

This dataset condenses a number of procedures that includes data collection, data analysis, data management and data publication. The result is an up to date, comprehensive and coherent view of species groups in a territory. This allows for many kinds of analyses.

Most of the procedures carried out are standard practices. However, we provide some references and details that we consider useful and to avoid errors in potential analyses.

We may group methods and procedures carried out as follows:

Field work and herbarium specimens study followed the traditional procedures (see, e.g., ref 21).

Specimens studied and species delimitation: 89 characters were scored for the species description: 58 qualitative and 27 quantitative traits, as well as four ratios. Ideally, at least 15 specimens were scored for each species. Quantitative characters were assessed with a Digital caliper Mitutoyo CD-15CD. The most frequent values are percentiles, and the extreme values are enclosed in parentheses. Scanning electron microscopy (SEM) of pollen, mericarps, and seeds were undertaken to assess better the species circumscription in some difficult cases. SEM samples were glued to aluminum stubs, coated with 40–50 nm gold, and examined with a JEOL TSM T330A scanning electron microscope at 15 kV.

Previous accounts on the genus have been revised. Among these the following are worth mentioning: the monograph by Knuth^22^, still the most complete treatment of the genus; Moore^23^, for species of Mexico and central America; Macbride^24^, for Peru and Barboza^25^, for Argentina. For a more detailed review of the taxonomic history of the genus see^19^.

Data processing. Details --measures, label facts, nomenclature, etc.-- from all studied specimens were entered into a MS-Access database designed and built for that purpose. Additional tables were created to record specimen measures as well as bibliographic, taxonomic and nomenclatural information --these are not included in this dataset, but nomenclature information is available through the ‘Catalogue of Life’^26^. Specific guidelines regarding data entry and quality control procedures followed^27,28^. The dataset was published in a standardized format using the Integrated Publishing Toolkit^29^ (IPT) that is hosted by GBIF Spain. The standardized format is Darwin Core Archive (DwC-A, GBIF^30^), which is a biodiversity data standard that makes use of Darwin Core terms^31^. A Darwin Core Archive is a zip file containing a data file in tab delimited text (.txt) format, a xml file describing the data file, and the relationships between the archive’s data files when there is more than one (meta.xml), and a machine readable dataset metadata in XML format (eml.xml), complying with GBIF Metadata Profile^32^ based on EML.

This is a curated dataset with a high quality of expertise, and updates are planned as new and relevant data is compiled. The IPT platform (http://www.gbif.es/ipt) serves as the dataset’s repository by archiving all published versions of the dataset and allowing changes to the dataset over time to be easily tracked.

## Data Records

This dataset is managed in a live database, and thus any errors are corrected as identified and taxonomic details updated as appropriate. It is accessible from the GBIF Spanish Node IPT platform (Data citation 1). This IPT platform (www.gbif.es/ipt) allows the download of data in DwC-A format, of metadata in EML^33^ and RTF formats, provides access to the different versions of the dataset in a controlled and documented way. Besides, this dataset is indexed by the GBIF portal (www.gbif.org, where it can be queried along with the thousands of datasets that constitute the GBIF data network in an integrated way. The GBIF data portal also provides discovery, download and view services via its API, and tags all downloads using DOIs, which support robust citation and traceability. The dataset is made available under a CC-BY 4.0 licence.

The authors would appreciate that any errors identified by users of the dataset be reported to the corresponding author to enable correction where necessary, allowing improvement for subsequent users.

Records are formatted following the Darwin Core specification^31^. Out of the 150 plus terms defined by the standard, 48 have been used in the dataset; these are enumerated in Table 2.

Some aspects to be noted here are:

Global Unique identifiers formatted as UUDIs^34^ are provided for each record (column occurrenceID).

Accepted, verified scientific names are provided in all cases. Higher classification follows Aedo^26^ and is congruent with APG IV^35^.

Specimens with some kind of specific nomenclatural status are marked accordingly using column ‘Typestatus’ values found there are: Epitype, Holotype, Isotype, Lectotype, Neotype or Paratype all in accordance with the ICBN^36^. In some cases, additional details on the specimen’s nomenclatural type status are given under the OccurerrrenceRemarks column.

Locality details are provided under columns country, stateProvince, and locality. Distribution status (introduced, native, naturalised, uncertain) under column establishmentMeans.

Geographic coordinates are given for 8,538 (95%) records with a 1-minute accuracy, (columns decimalLatitude, decimalLongitude, geodeticDatum, coordinatePrecision, coordinatePrecision).

Habitat information is provided for all newly collected specimens, but only available for a fraction of the material studied in herbaria, and as a result, it is only provided for 3,952 (44%) records under column ‘habitat’.

Collections where specimens are deposited are indicated by their ‘Index Herbariorum’^37^ acronyms in column ‘otherCatalogNumbers’. Records containing information of duplicate specimens have been aggregated in one record, and acronyms of herbaria where duplicated are deposited are also indicated in column ‘otherCatalogNumbers’.

## Technical Validation

Dataset publication in the GBIF network include data transformation to comply with the Darwin Core specification^31^ and uploaded onto the GBIF-Spain IPT (http://www.gbif.es/ipt/). The IPT guarantees the outputted data files are valid, meaning they are standardized correctly and are valid XML, and also ensures each species occurrence record has a unique identifier and basisOfRecord.

Validation procedures (geographic coordinate format, coordinates within country/provincial boundaries, absence of ASCII anomalous characters in the dataset) were performed with DARWIN_TEST (v3.3) software^38^, and additional checks were performed using the Vesper platform^39^.

Furthermore, the data has been successfully indexed into GBIF.org, that also implies a number of validations such as checking that each species occurrence record has a unique identifier and a basisOfRecord, complying with the Darwin Core Type Vocabulary, spatial checks, date checks, etc. The index process produces a report available to all users.

**Figure 3:**
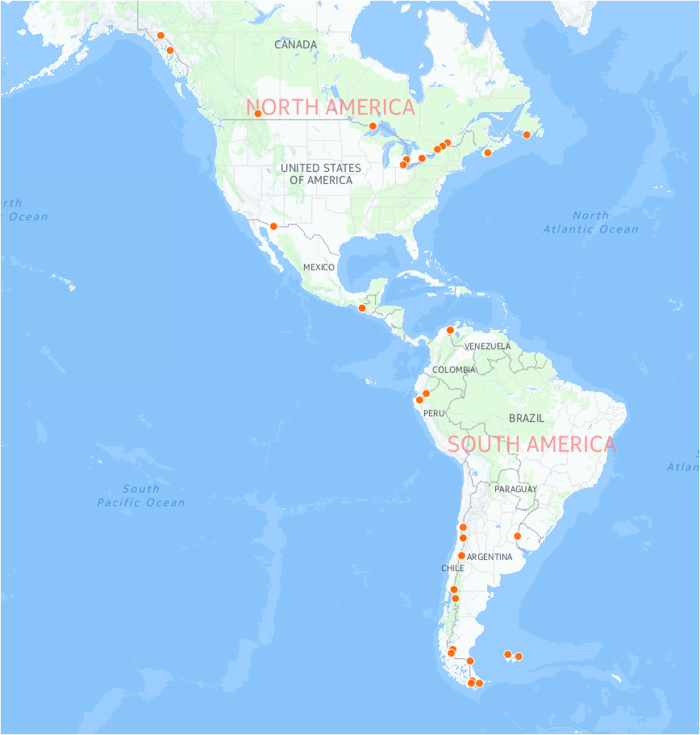
Map showing records with an apparent mismatch between coordinates and country.

The issues on dates correspond to cases where the collecting or recording of day and/or month was missing. We opted for keeping the available information even though the result was not-well-formed date strings. As published, country/coordinate mismatch issues were flagged in 50 cases, these correspond to records located very close to a country border and fall with the acceptable imprecision (100 s m in most cases, a few Km in a small number of cases), and bear consequences for all but the most local analyses. Figure 3 shows the location of these apparent mismatches.

## Usage Notes

Since this dataset is indexed by the GBIF portal (www.gbif.org) it can be queried along with thousands of other datasets --all normalised under the same standards-- and thus allowing visualisation and analysis in an integrated way. Besides, a growing plethora of software tools further facilitates data visualization and exploitation of GBIF’s network datasets (e.g. R packages such as ‘rgbif’^40^ or ‘dismo’^41^, or ‘ModestR’^42^).

However, as it is always the case when aggregating taxonomic data, species concepts coming from different datasets should be carefully scrutinised for compatibility and consistency, as to avoid working with chimera species concepts that will result inevitably in wrong conclusions and results. Species concepts used in this dataset are congruent with those in Catalogue of Life, as the expert behind is the same^26^. Explicit descriptions of the species contained in the dataset are available in works previously cited^11–19^. Coordinates for georeferenced records are given with a 1-minute accuracy.

## Additional Information

**How to cite this article**: Aedo, C. & Pando, F. A distribution and taxonomic reference dataset of Geranium in the New World. *Sci. Data* 4:170049 doi: 10.1038/sdata.2017.49 (2017).

**Publisher’s note:** Springer Nature remains neutral with regard to jurisdictional claims in published maps and institutional affiliations.

## Supplementary Material



## Figures and Tables

**Table 1:** The top ten collections by number of studied specimens and number of specimens studied in each one.

**Herbarium**	**NO. of studied specimens**
Missouri Botanical Garden. St Louis. USA (MO)	1,493
The New York Botanical Garden. USA (NY)	1,181
Real Jardín Botánico. Madrid. Spain (MA)	774
Harvard University. Cambridge. USA (GH)	666
Field Museum of Natural History. Chicago. USA (F)	630
Universidad Nacional Autónoma de México. D.F. México (MEXU)	504
Universidad Nacional. Bogotá. Colombia (COL)	469
Agriculture and Agri-Food Canada. Ottawa. Canada (DAO)	440
Universidad de Concepción. Concepción. Chile (CONC)	413
The Natural History Museum. London. UK (BM)	258

**Table 2:** Darwin Core terms^31^ used in the dataset.

**Columns**
Name
occurrenceID
institutionCode
collectionID
collectionCode
catalogNumber
kingdom
phylum
class
order
family
genus
subgenus
specificEpithet
infraspecificEpithet
scientificName
scientificNameAuthorship
taxonRank
dateIdentified
identifiedBy
typeStatus
continent
country
stateProvince
locality
decimalLatitude
decimalLongitude
geodeticDatum
coordinatePrecision
coordinateUncertaintyInMeters
minimumElevationInMeters
maximumElevationInMeters
basisOfRecord
eventDate
year
month
day
habitat
fieldNumber
recordedBy
samplingProtocol
otherCatalogNumbers
establishmentMeans
occurrenceRemarks
occurrenceStatus
preparations
associatedReferences
nomenclaturalCode
taxonomicStatus
